# Stylus Tip Center Position Self-Calibration Based on Invariable Distances in Light-Pen Systems

**DOI:** 10.3390/s17010131

**Published:** 2017-01-11

**Authors:** Rui Zhang, Shugui Liu, Sen Wang, Xuanxiao Song

**Affiliations:** State Key Laboratory of Precision Measuring Technology and Instruments, Tianjin University, Tianjin 300072, China; sgliu@tju.edu.cn (S.L.); wang.sen.happy@163.com (S.W.); xuanxiao_song@126.com (X.S.)

**Keywords:** light-pen CMM, tip center position, self-calibration, invariable distances, kinematic seat with an inverted cone hole

## Abstract

The light-pen coordinate measuring machine (LPCMM for short) is portable and flexible to measure features including invisible ones in-situ. Since different styluses are needed to measure different features and even during the process of measuring a single workpiece with complicated configurations, to improve the system measurement accuracy it is beneficial to calibrate the stylus tip center position after it is mounted to the light-pen before measurement in an industrial field. A novel and simple method aiming at self-calibrating the position of the tip center based on invariable distances is presented. The distinguishing feature of the proposed method is that the center position of the tip can be calibrated by using a kinematic seat with an inverted cone hole without any external reference and auxiliary devices. Calibration is based on that the distance between the tip center and that of any LED is invariable when the light-pen is swung smoothly with its spherical tip firmly touching the fixed cone seat. To ensure the repeatability of the algorithm some error constraint parameters are given. Based on invariable distances, the tip center position in the light-pen coordinate system can be obtained. Experiment results show that the self-calibration method has the advantage of good repeatability, with standard deviations 0.027, 0.023 and 0.014 mm in *U*, *V* and *W* directions, respectively. Experimental results of measuring a circle and a gauge block indirectly demonstrate the accuracy of the proposed self-calibration method.

## 1. Introduction

As a kind of mature and high-precision measuring instrument, coordinate measuring machines (CMMs for short) have gained wide acceptance in many fields, such as mechanical manufacturing, aviation, reverse engineering, etc. Different types of physical quantities, such as length, diameter, roundness and many others, can be obtained with CMMs by measuring points on the surface of workpieces. However, the most widely used Cartesian CMMs are not portable and cannot meet the requirements of in-situ measurement because of their large size and heavy weight [1,2]. During the past few decades, to meet the need of in-situ measurement in the field and especially in large-scale metrology [3], there has been a focus on portable and flexible measuring techniques, even at the cost of sacrificing accuracy to a certain degree. Many types of novel portable coordinate measuring machines (PCMMs for short) such as articulated arm CMM, laser tracker, indoor GPS, mobile spatial CMM, photogrammetric system, have seen broad and rapid development and brought great convenience to in-situ and large-scale measurement tasks [4,5,6,7,8]. 

The light-pen coordinate measuring machine (LPCMM for short) uses a light-pen as its detecting element. It mainly consists of a light-pen, one or two industrial cameras, a laptop computer and associated software. Some other auxiliary devices may be attached to enhance its function. The light-pen is a kind of handheld detection device with several mounted LEDs providing control points and a replaceable stylus. During the measurement, the stylus tip of the light-pen touches the workpiece surface firmly and the position of the measured point is determined by the images of control points, which are called feature points, and their coordinates are calculated through image processing algorithms. Since not the stylus tip but control points are imaged, even hidden features, such as small and deep holes, invisible points or corners inside the measured piece, which cannot be sensed by optical PCMMs, can be measured by properly selecting the stylus [9,10,11,12].

Since only the positions of the control points are sensed by the camera, it is essential to know the relative position of the measured point with reference to the positions of control points. For this both the diameter of the spherical tip and the center position of the tip in the light-pen coordinate system, as well as the camera system, should be accurately calibrated. During the past several decades there have been a large number of studies on the topic of camera calibration, such as Tsai’s two-stage approach [13], Zhang’s method using 1D, 2D or 3D reference objects [14,15], the bundle adjustment method [16], the direct linear transformation method [17] and many others [18,19]. By contrast, very few publications about the calibration of the light-pen could be found. The tip diameter is fixed for a stylus and will not be changed with remounting so it can be calibrated in advance by using a reference ball or a gauge block in the lab and recalled while knowing which stylus is mounted on the light-pen. Since it is a common practice [1], we will simply discuss it and will concentrate our discussion on the tip center position calibration.

System calibration, including the camera calibration, center position calibration of control points and the tip, should be completed before measurement. The center position calibration of control points can be completed in a temperature controlled laboratory environment with high-precision equipment, such as CMMs, since for a well-designed light-pen their positions will not be changed during transportation, mounting and use. However, the center position of the stylus tip center cannot be the same when different kinds of stylus are mounted to the light-pen. In some cases, several different styluses may be applied to complete a single in-situ measurement. After a stylus is replaced by another one, the tip center position is changed and an in-situ calibration is required before further measurement can be performed. Furthermore, to protect the styluses from damage, they should be placed in a special box when not in use, and mounted to the light-pen before usage, so the tip center position needs to be calibrated in-situ rather than pre-calibrated in a lab [20]. The approach introduced in [21] needs to take two groups of images and consists of three calibration steps. More-over, the standard deviations of its repeatability test reach (0.178, 0.188, 0.221) (mm) in *x*, *y* and *z* directions, respectively and cannot meet the requirement of accurate measurement. The methodology introduced in [22] has an advantage of the relatively high repeatability with standard deviations (0.033, 0.030, 0.043) (mm) in *x*, *y* and *z* directions, respectively. However, its algorithm has the big problem that it does not converge sometimes.

To calibrate the tip center position in-situ, the method should be as simple as possible. It is preferred not to rely on a high accurate reference. A novel method for self-calibration of the tip center position with high accuracy based on invariable distances is presented in the paper. In Section 2, the system structure and model of the LPCMM is presented. The self-calibration model and calibration steps of the tip center position are discussed in Section 3. Repeatability tests and several measurement experiments are presented in turn in Section 4. Some conclusions are given in Section 5. 

## 2. Light-Pen Coordinate Measurement System

### 2.1. System Structure of the LPCMM

**Figure 1:**
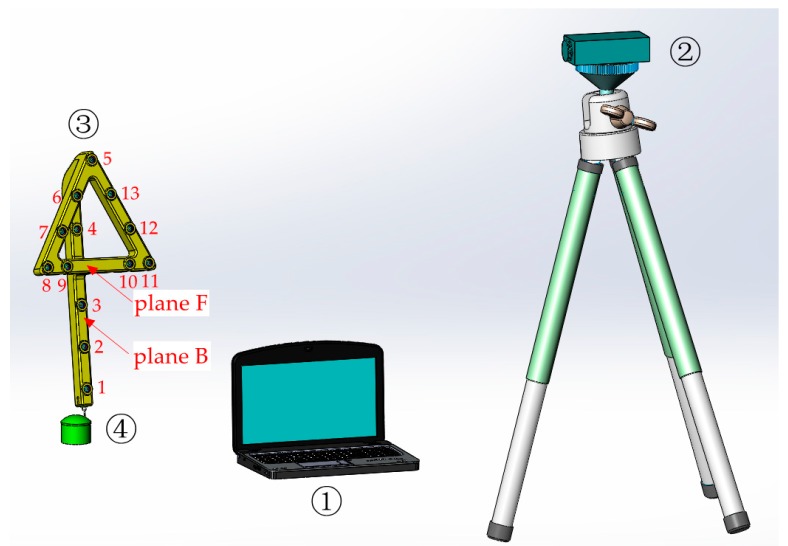
The LPCMM system. ① Laptop computer, ② camera, ③ light-pen, ④ workpiece.

Figure 1 shows the whole system structure of the LPCMM. This system is mainly composed of a laptop computer, a camera and a light-pen. Because more information can be obtained from images of the light-pen in the dual-camera LPCMM, theoretically, it has a higher measurement accuracy than the single-camera one. However, the system will be more complicated and the calibration for the relative position between two cameras as well as the limited measured area make the dual-camera LPCMM not so flexible in an industrial field. Only the single-camera system will be discussed in this paper.

**Figure 2:**
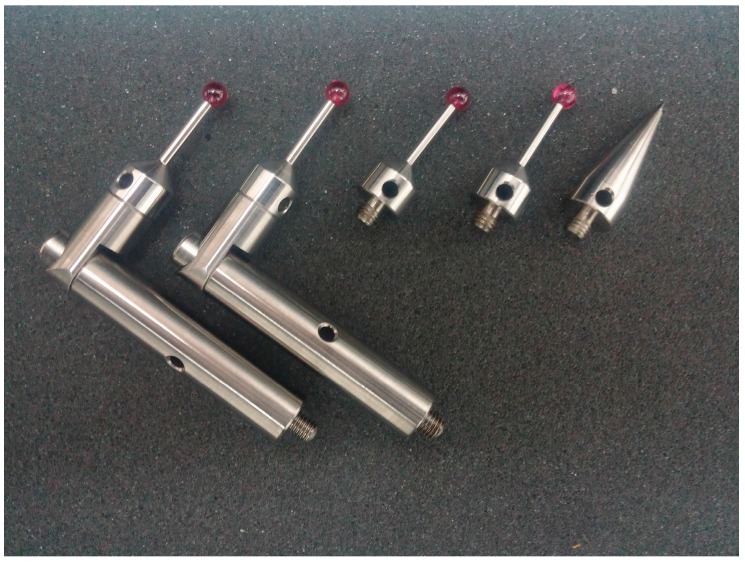
Light-pen styluses.

As a type of handheld detection device, the light-pen is presented in Figure 1. The body of the light-pen, made of carbon fiber, is rigid, of light weight and almost free from temperature influences. To meet different measurement requirements, such as measuring different shapes and sizes of deep holes, the stylus of the light-pen should be replaceable. Several types of styluses are shown in Figure 2. In order to capture stable images with suitable brightness of feature points, high power infrared LEDs are chosen as control points. They can emit the light with a wavelength of about 870 nm. As a suitable light filter is mounted to the camera lens, the stray background light will have little impact on the measurement. To further improve the measurement repeatability and accuracy, not only each control point could be switched on or off separately, but also the lightening time spans and brightness of the LEDs can be dynamically controlled in real time, according to environmental changes [23]. That means with this dynamic adjustment algorithm, the repeatability of feature point center locations will not be influenced by the distance between the camera and the light-pen to certain extent. Thirteen LEDs are arranged as shown in Figure 1: Nine of them are distributed uniformly in the front plane (plane F), and other four are arrayed evenly in a line in the back plane (plane B), which is parallel to the front plane at a distance of 100 mm. 

### 2.2. Coordinate System Establishment of the LPCMM

**Figure 3:**
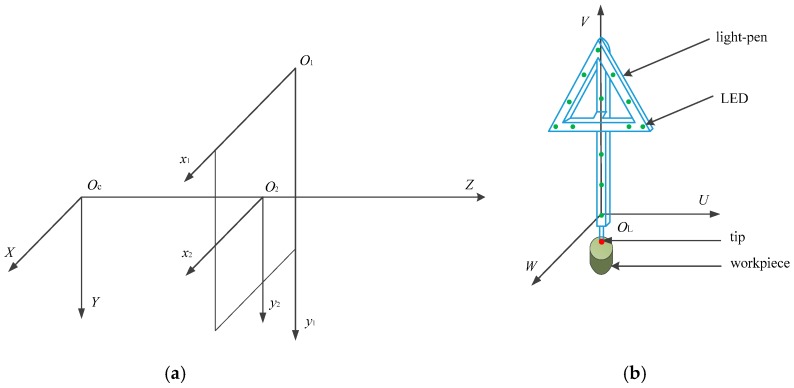
Four coordinate systems of the LPCMM. (**a**) Coordinate systems in the camera; (**b**) Coordinate system in the light-pen.

There are four coordinate systems in the LPCMM, as shown in Figure 3:

(a) The light-pen coordinate system *O*_L_-*UVW*. The origin *O*_L_ is set at the center of the LED 1 (marked in Figure 1). The axis *U* is parallel to the line connecting LEDs 8 and 11, and its positive direction is towards LED 11. The axis *V*, perpendicular to *U*, is parallel to the line linking LEDs 1 and 4, and its positive direction is towards LED 4. The axis *W* is set up according to the right-hand rule.
(b) The pixel coordinate system *O*_1_*-x*_1_*y*_1_. The origin is placed at the up-right corner of the image plane. *x*_1_ and *y*_1_ are parallel to the horizontal and vertical pixel arrays, respectively. The orientations of *x*_1_ and *y*_1_ are built to make all coordinate values of the pixels positive.
(c) The image-plane coordinate system *O*_2_-*x*_2_*y*_2_. *O*_2_ is defined at the intersection point of the optical axis of the camera with the image plane. *x*_2_ and *y*_2_ are parallel to *x*_1_ and *y*_1_, respectively.
(d) The camera coordinate system *O*_C_-*XYZ*. *O*_C_ is placed at the perspective center of the camera. *X* and *Y* are parallel to *x*_1_ and *y*_1_, respectively. *Z* is the optical axis of the camera with positive direction from *O*_C_ to *O*_2_.

### 2.3. System Model of the LPCMM

During the measurement, the stylus tip of the light-pen should touch the workpiece surface steadily and keep the light-pen’s body vertically with LEDs facing the camera. Photos of the light-pen are taken by the camera and make sure that all 13 LEDs are well imaged to feature points. With algorithm processing, these coordinates of the center of feature points in *O*_1_-*x*_1_*y*_1_, (*x*_1*i*_, *y*_1*i*_) (*i* = 1~13), are obtained:

(1) $$\left[\begin{array}{c} x_{2i}\\ y_{2i}\\ 1 \end{array}\right]=\left[\begin{array}{ccc} 1 & 0 & \delta_{x}\\ 0 & 1 & \delta_{y}\\ 0 & 0 & 1 \end{array}\right]\left[\begin{array}{ccc} d_{x} & 0 & 0\\ 0 & d_{y} & 0\\ 0 & 0 & 1 \end{array}\right]\left[\begin{array}{ccc} 1 & 0 & -c_{x}\\ 0 & 1 & -c_{y}\\ 0 & 0 & 1 \end{array}\right]\left[\begin{array}{c} x_{1i}\\ y_{1i}\\ 1 \end{array}\right]$$

In Equation (1), (*x*_2*i*_, *y*_2*i*_) are corresponding coordinates of (*x*_1*i*_, *y*_1*i*_) in *O*_2_-*x*_2_*y*_2_. (*δ_x_*, *δ_y_*, *d_x_*, *d_y_*, *c_x_*, *c_y_*) are the intrinsic parameters of the camera, and can be obtained by the sub-regional camera calibration method given in [17]. (*δ_x_*, *δ_y_*) are distortion values of the camera lens in *x*_1_ and *y*_1_ directions respectively. (*d_x_*, *d_y_*) are the sizes of the pixel in in *x*_1_ and *y*_1_ directions separately, while (*c_x_*, *c_y_*) are the position of the point *O*_2_ in *O*_1_-*x*_1_*y*_1_:

(2) $$\left[\begin{array}{c} x_{i}\\ y_{i}\\ z_{i} \end{array}\right]=\left[\begin{array}{c} x_{2i}\\ y_{2i}\\ f \end{array}\right]$$

where *f* is the focus length of the camera. (*x_i_*, *y_i_*, *z_i_*) are coordinates of feature point centers in *O*_C_-*XYZ*.

From Equations (1) and (2), (*x_i_*, *y_i_*, *z_i_*) can be calculated:

(3) $$\left[\begin{array}{c} x_{i}^{\prime }\\ y_{i}^{\prime }\\ z_{i}^{\prime } \end{array}\right]=\left[\begin{array}{ccc} \frac{z_{i}^{\prime }}{z_{i}} & 0 & 0\\ 0 & \frac{z_{i}^{\prime }}{z_{i}} & 0\\ 0 & 0 & \frac{z_{i}^{\prime }}{z_{i}} \end{array}\right]\left[\begin{array}{c} x_{i}\\ y_{i}\\ z_{i} \end{array}\right]$$

where (*x_i_*’, *y_i_*’, *z_i_*’) are coordinates of the LED centers in *O*_C_-*XYZ*.

Since the coordinates of the LED centers in *O*_L_-*UVW*, (*u_i_*, *v_i_*, *w_i_*) have been pre-calibrated by a high-accurate CMM in the lab, the rotation matrix ***R*** given in Equation (5) or (6) and the translation vector ***T*** (*t_x_*, *t_y_*, *t_z_*) could be computed from Equations (3), (4) and (6) by using the nonlinear least square generalized inverse method given in [24]:

(4) $$\left[\begin{array}{c} x_{i}^{\prime }\\ y_{i}^{\prime }\\ z_{i}^{\prime } \end{array}\right]=\left[\begin{array}{cccc} r_{1} & r_{2} & r_{3} & t_{x}\\ r_{4} & r_{5} & r_{6} & t_{y}\\ r_{7} & r_{8} & r_{9} & t_{z} \end{array}\right]\left[\begin{array}{c} u_{i}\\ v_{i}\\ w_{i}\\ 1 \end{array}\right]$$

where *r_i_* (*i* = 1~9) and (*t_x_*, *t_y_*, *t_z_*) are the parameters of the rotation and translation matrices between the *O*_L_-*UVW* and *O*_C_-*XYZ*, respectively. *r_i_* in Equation (4) are functions of rotated angles *α*, *β* and *γ* as shown in Equation (5):

(5) $$R=\left[\begin{array}{ccc} \cos\beta \cos\gamma & \sin\alpha \sin\beta \cos\gamma -\cos\alpha \sin\gamma & \cos\alpha \sin\beta \cos\gamma +\sin\alpha \sin\gamma \\ \cos\beta \sin\gamma & \sin\alpha \sin\beta \sin\gamma +\cos\alpha \cos\gamma & \cos\alpha \sin\beta \sin\gamma -\sin\alpha \cos\gamma \\ -\sin\beta & \sin\alpha \cos\beta & \cos\alpha \cos\beta \end{array}\right]$$

where *α*, *β* and *γ* are the rotation angles around *U*, *V* and *W* axes, respectively. The rotations are in the order of *α*, *β* and *γ*. When the light-pen is almost vertical, (*α*, *β*, *γ*) equal to (π, 0, 0) approximately, ***R*** could be simplified as shown in Equation (6):

(6) $$R\approx \left[\begin{array}{ccc} 1 & \gamma & -\beta \\ \gamma & -1 & \alpha -\pi \\ -\beta & \pi -\alpha & -1 \end{array}\right]$$

When coordinates of the tip center in system *O*_L_-*UVW*, (*u*_0_, *v*_0_, *w*_0_), are known, corresponding coordinates in system *O*_C_-*XYZ*, (*x*_0_, *y*_0_, *z*_0_), can be calculated from Equation (7):

(7) $$\left[\begin{array}{c} x_{0}\\ y_{0}\\ z_{0} \end{array}\right]=\left[\begin{array}{cccc} r_{1} & r_{2} & r_{3} & t_{x}\\ r_{4} & r_{5} & r_{6} & t_{y}\\ r_{7} & r_{8} & r_{9} & t_{z} \end{array}\right]\left[\begin{array}{c} u_{0}\\ v_{0}\\ w_{0}\\ 1 \end{array}\right]$$

**Figure 4:**
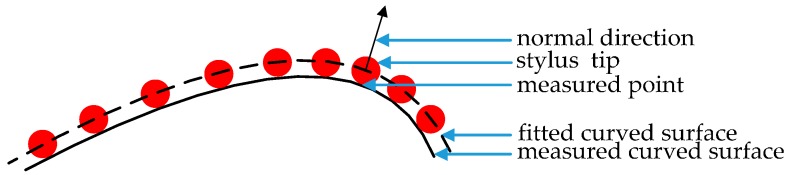
Tip radius compensation.

The coordinates of the measured point in *O*_C_-*XYZ* can be obtained with tip radius compensation. As shown in Figure 4, the enveloping surface method is always used to compensate the tip radius. The fitted curved surface could be obtained with center positions of the stylus tip. Then the measured point’s position in *O*_C_-*XYZ* can be obtained by the corresponding stylus tip center position in *O*_C_-*XYZ*, its normal direction in *O*_C_-*XYZ* and the tip radius.

The tip center position varies when the stylus is changed and mounted, even when the same stylus is remounted. Self-calibration of (*u*_0_, *v*_0_, *w*_0_) in-situ is essential.

## 3. Self-Calibration of the Tip Center Position

### 3.1. Establishment of the Self-Calibration Model

**Figure 5:**
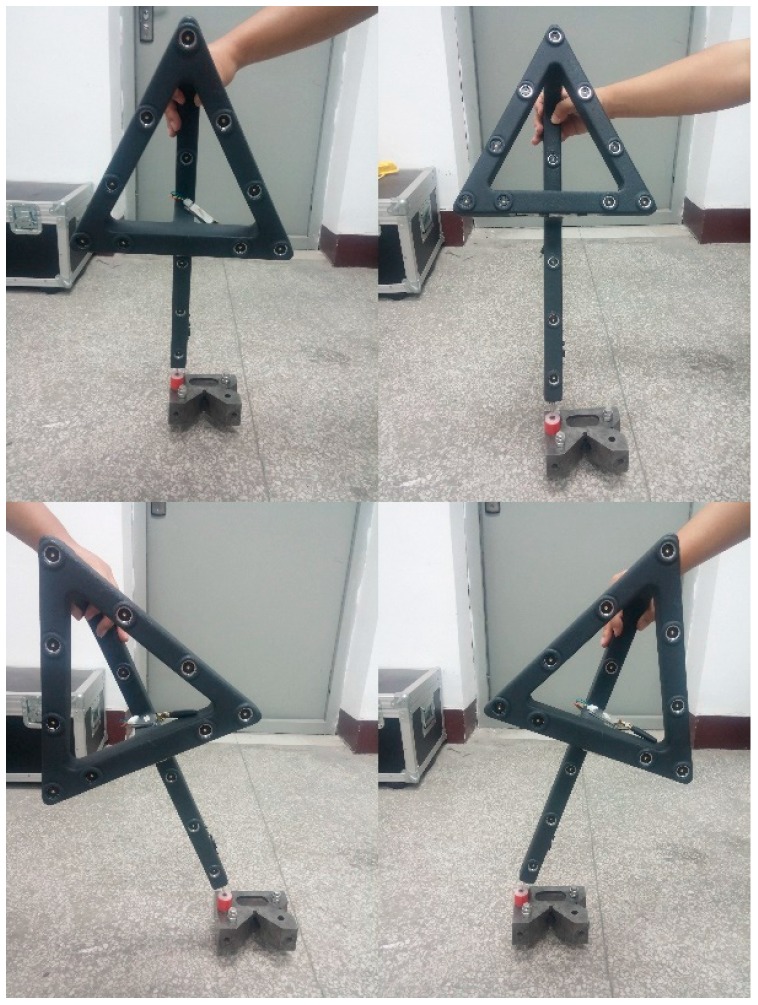
Four gestures for self-calibration.

As shown in Figure 5, the stylus tip of the light-pen should be kept closely and firmly in touch with the fixed kinematic seat with an inverted cone hole during self-calibration. The camera is fixed at a suitable position so all LEDs always appear in the camera’s field of view. Then we swing the light-pen smoothly and slowly with all LEDs facing the camera.

As known from above, the coordinates of the tip center are (*u*_0_, *v*_0_, *w*_0_) in *O*_L_-*UVW* which needs to be calibrated. The light-pen can be treated as a rigid body, (*u*_0_, *v*_0_, *w*_0_) are unchanged when the light-pen swings. Its coordinates, (*x*_0_, *y*_0_, *z*_0_), in *O*_C_-*XYZ* are constants as well, because the relative position between the kinematic seat and the camera is invariable and the spherical tip touches the cone hole firmly during calibration.

(a) After all parameters of the matrices ***R*** and ***T*** are determined in accordance with the equations given in Section 2.3. Several images of control points are taken and determined ***R*** and ***T*** are verified by calculating the reprojection error [25,26,27] as follows:
  (8) $$\Delta p_{j}=\sum_{i=1}^{13}\sqrt{{\left(x_{2i}^{\prime }-x_{2i}\right)}^{2}+{\left(y_{2i}^{\prime }-y_{2i}\right)}^{2}}$$
  The re-projected feature points (*x*_2*i*_’, *y*_2*i*_’) (*i* = 1~13) are obtained from pre-calibrated (*u_i_*, *v_i_*, *w_i_*) and the calculated matrices ***R***, ***T***. (*x_ij_*″, *y_ij_*″, *z_ij_*″) are defined from Equation (9) given below. *j* is the serial number of image. For assuring the required accuracy of calibration it is suggested to take at least seven images.
  It is known that the center positions of feature points can be determined more accurately when the light-pen is vertical than it is slant. When the pitch angle of the light-pen is small during the calibration, Δ*p_j_* will be small. However, the variations of (*x_ij_*″, *y_ij_*″, *z_ij_*″) will be small as well, and it will lead to large errors in solving the equations. Conversely, when the pitch angle of the light-pen is big, Δ*p_j_* will be big too. So two threshold values, *Q*_1_ < *Q*_2_, should be given for Δ*p_j_* to make the pitch angle of the light-pen within a suitable range and to obtain eligible parameters of the matrices ***R***, ***T***.
(b) In *O*_C_-*XYZ*, the distance between the center of each LED and the tip center *d_i_* (*i* = 1~13), and (*x*_0_, *y*_0_, *z*_0_) can be determined by solving the following equation using the nonlinear least square generalized inverse method:
  (9) $${\left(x_{ij}^{\prime \prime }-x_{0}\right)}^{2}+{\left(y_{ij}^{\prime \prime }-y_{0}\right)}^{2}+{\left(z_{ij}^{\prime \prime }-z_{0}\right)}^{2}={d_{i}}^{2}$$
  where (*x_ij_*″, *y_ij_*″, *z_ij_*″) are coordinates of the center of the *i*-th LED in *O*_C_-*XYZ* system in the *j*-th image.
(c) Because of unavoidable errors in calibration, the distance between the center of the *i*-th LED and the center of the probe tip cannot be the same as *d_i_* calculated from Equation (9). Δ*d_j_* of the *j*-th image is the sum of the absolute values of the differences between these two distances in the *j*-th image. Two threshold values *Q*_3_, *Q*_4_ are given for Δ*d*_max_, the maximum of all Δ*d_j_*, and variation of Δ*d*_max_ defined in Equation (10):
  (10) $$\begin{array}{l} \Delta d_{j}=\sum_{i=1}^{13}\left|\sqrt{{\left(x_{ij}^{\prime \prime }-x_{0}\right)}^{2}+(y_{ij}^{\prime \prime }-y_{0}{)}^{2}+(z_{ij}^{\prime \prime }-z_{0}{)}^{2}}-d_{i}\right|\\ \Delta d_{\max}\;=\;\max(\Delta d_{j}) \end{array}$$
(d) As the distances, *d_i_* (*i* = 1~13), are invariable in both *O*_L_-*UVW* and *O*_C_-*XYZ*, all 13 equations shown in Equation (11) can be solved in the same way as in *O*_L_-*UVW*:
  (11) $${\left(u_{i}-\;u_{0}\right)}^{2}+{\left(v_{i}-v_{0}\right)}^{2}+{\left(w_{i}-w_{0}\right)}^{2}={d_{i}}^{2}\;\left(i=\text{1\textasciitilde{}13}\right)$$

In this way, the stylus tip center position of the light-pen in *O*_L_-*UVW*, (*u*_0_, *v*_0_, *w*_0_), can be self-calibrated.

### 3.2. Calibration Steps

(a) To process one of the image and calculate the matrices ***R****_j_*, ***T****_j_* from Equations (1)–(6) and then to determine (*x*_2*i*_’, *y*_2*i*_’) and (*x_ij_*″, *y_ij_*″, *z_ij_*″) (*i* = 1~13, *j* ≥ 7) from Equations (2)–(4). To compute Δ*p_j_* in Equation (8) and save the parameters of ***R****_j_*, ***T****_j_* if *Q*_1_ < Δ*p_j_* < *Q*_2_. Otherwise ignore this photo.
(b) After at least seven eligible images are obtained, 13 distances *d_i_* (*i* = 1~13) and (*x*_0_, *y*_0_, *z*_0_) can be determined from Equation (9).
(c) To calculate Δ*d*_max_ from Equation (10) and remove the image in which Δ*d_j_* is the biggest, and then to capture one more image and go back to step (a). After that, a new Δ*d*_max_ is obtained. If this new Δ*d*_max_ < *Q*_3_ and the difference between two Δ*d*_max_ obtained from adjacent seven eligible photos is less than *Q*_4_, then distances *d_i_* (*i* = 1~13) are considered as valid ones. Otherwise, similarly, to give up the image in which Δ*d_j_* is the biggest, and to capture one more image and go back to step (a).
(d) To determine the coordinates of the tip center in *O*_L_-*UVW*, (*u*_0_, *v*_0_, *w*_0_) from Equation (11) based on eligible *d_i_*.

## 4. Experiments

Repeatability tests of tip center position self-calibration at different distances and experiments for measuring a circle and a gauge block have been carried out in a lab environment with the temperature about 20 °C. 

### 4.1. Repeatability Tests of Tip Center Position Self-Calibration 

By using the method mentioned above, the tip center positon of the light-pen has been self-calibrated ten times to test the repeatability of the method proposed in this paper. The test results are given in Table 1.

As shown in Table 1, the average value (Ave for short), the standard deviation (Std for short) and the range (the difference between the maximum and minimum value) of the calibrated tip center positon, as well as the total number of photos needed for particular calibration, are presented. It can be seen that the dispersion range is (0.085, 0.068, 0.045) (mm) with the standard deviation (0.027, 0.023, 0.014) (mm) in the *U*, *V* and *W* directions, respectively. It shows the repeatability of the self-calibration method based on invariable distances could meet the needs of measurement systems with submillimeter accuracy. The total number of photos cannot be the same during each calibration, for that the number will be affected by many factors, such as the proficiency of experiment operator and some others. However, less than 300 photos are needed and it takes less than 2 min for each calibration, which shows that the proposed method of self-calibration has the advantage of high efficiency. 

During the calibration, the distance between the camera and the kinematic seat or the light-pen should be suitable. If it is too short, brightness of feature points is too high to obtain their center positions accurately, even with the dynamic adjustment algorithm of feature points, whereas if the distance is too long, the solution accuracy for the LPCMM system will decrease. More images are needed for the self-calibration described above with the increase of the distance. In the repeatability experiment above, the camera keeps about 1.4 m away from the kinematic seat. However, this distance cannot be the same in each practical self-calibration. Table 2 shows the results of self-calibration at different distances. The average values, the standard deviations and the ranges of ten tests for each distance are listed. In Table 2, the dispersion ranges of the average *u*_0_, *v*_0_, *w*_0_ at different distances are (0.084, 0.108, 0.094) (mm) with the standard deviations (0.030, 0.035, 0.042) (mm) in *U*, *V* and *W* directions, respectively, which is acceptable. From the table, it can be seen that the absolute values of *u*_0_ and *v*_0_ become bigger and bigger as the distance increases, while the value of *w*_0_ has no significant changes. It might be caused by the error in camera focal length calibration. Some deep studies are needed.

### 4.2. Measurement Experiments of the System with the (U_0_, V_0_, W_0_) Self-Calibrated

**Figure 6:**
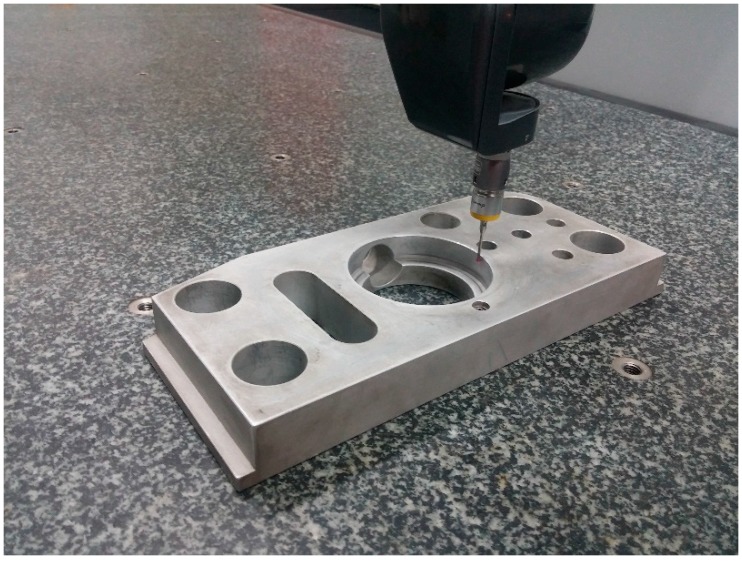
The measured circle.

In order to test the accuracy of the self-calibration method based on invariable distances, several measurement experiments are performed. A circle shown in Figure 6 and a gauge block were measured with the self-calibrated values of (*u*_0_, *v*_0_, *w*_0_) equal to (−1.988, −70.663, −16.636) (mm) in the *U*, *V*, *W* directions, respectively.

The diameter of the circle measured by the CMM equals to 63.521 mm, taken as its true value. The maximum permissible error (MPE for short) of the CMM is 2.3 + 3.3 *L*/1000 (μm) where *L* is the measured length in mm. The circle was measured at five different positions and ten times at each position while the distance between the camera and the light-pen changes from 1.5 m to 9 m. The test results are given is Table 3. The standard deviation and the dispersion range increase with the increase of distance. The maximum absolute error (Abs for short) is 0.083 mm, which indirectly proves the validity of the self-calibration method.

Similar experiments have been performed by measuring a gauge block of 500 mm with uncertainty ±2.20 μm. The experimental results are shown in Table 4. The largest error in its absolute value is −0.119 mm measured at distance of 1.5 m. The length of the gauge block is obtained by measuring several points on its two end surfaces. The images of these points are near the boundary of the image plane, where the distortion errors are quite large even after corrections. However, the maximum absolute error is still acceptable, that indirectly verifies the validity of the self-calibration method again.

## 5. Conclusions

A new convenient method for stylus tip center position self-calibration based on invariable distances in the LPCMM is presented in this paper. Only a kinematic seat with an inverted cone hole is used and several images of control points are required to take during the self-calibration. The system structure and model of the whole system are given. The model and steps of self-calibration are discussed in detail. To verify the effectiveness of the proposed method repeatability tests at different distances and experiments by measuring a circle and a gage block have been carried out. Test results are satisfactory and show that the LPCMM system with the calibrated light-pen could realize submillimeter in-situ measurements. Further studies will be carried out in the real industrial field to apply it in practice.

## Figures and Tables

**Table 1:** Repeatability test of self-calibration for (*u*_0_, *v*_0_, *w*_0_).

Test	*u*_0_ (mm)	*v*_0_ (mm)	*w*_0_ (mm)	Photos Needed
1	−2.019	−70.639	−16.643	180
2	−1.964	−70.620	−16.657	158
3	−2.017	−70.678	−16.629	230
4	−1.970	−70.671	−16.655	206
5	−1.991	−70.633	−16.617	162
6	−1.988	−70.658	−16.636	158
7	−1.973	−70.683	−16.612	115
8	−1.953	−70.685	−16.634	132
9	−1.966	−70.688	−16.645	111
10	−2.037	−70.675	−16.632	276
Ave	−1.988	−70.663	−16.636	
Std	0.027	0.023	0.014	
Range	0.085	0.068	0.045	

**Table 2:** Results of self-calibration for (*u*_0_, *v*_0_, *w*_0_) at different distances (mm).

Distance	Ave			Std			Range		
	*u*_0_	*v*_0_	*w*_0_	*u*_0_	*v*_0_	*w*_0_	*u*_0_	*v*_0_	*w*_0_
1.2 m	−1.974	−70.633	−16.648	0.008	0.029	0.037	0.020	0.063	0.085
1.4 m	−1.988	−70.663	−16.636	0.027	0.023	0.014	0.085	0.068	0.045
1.6 m	−1.991	−70.678	−16.554	0.015	0.018	0.003	0.037	0.039	0.008
1.8 m	−2.011	−70.682	−16.559	0.040	0.015	0.040	0.094	0.035	0.098
2.0 m	−2.059	−70.741	−16.559	0.042	0.066	0.058	0.090	0.163	0.136
Std	0.030	0.035	0.042						
Range	0.084	0.108	0.094						

**Table 3:** Repeatability test for the circle diameter (in mm) measured at different distances.

Distance	1.5 m	3 m	5 m	7 m	9 m
1	63.595	63.603	63.550	63.605	63.427
2	63.633	63.608	63.658	63.627	63.338
3	63.636	63.679	63.582	63.546	63.324
4	63.602	63.673	63.474	63.619	63.744
5	63.616	63.548	63.584	63.627	63.625
6	63.576	63.549	63.629	63.489	63.562
7	63.594	63.583	63.588	63.603	63.486
8	63.584	63.616	63.560	63.470	63.489
9	63.590	63.560	63.516	63.412	63.553
10	63.617	63.555	63.619	63.729	63.355
Ave	63.604	63.597	63.576	63.573	63.490
Abs	0.083	0.076	0.055	0.052	−0.031
Std	0.019	0.046	0.051	0.089	0.128
Range	0.059	0.131	0.184	0.317	0.420

**Table 4:** Repeatability tests of measuring the length of a gauge block (in mm) at different distances.

Distance	1.5 m	3 m	5 m	7 m	9 m
1	499.840	499.965	500.048	500.087	499.927
2	499.874	499.960	500.036	500.127	500.048
3	499.871	499.949	499.995	500.080	499.948
4	499.919	499.960	500.035	499.993	499.894
5	499.888	499.946	500.025	500.073	499.873
6	499.900	499.921	500.037	499.965	499.925
7	499.847	500.003	500.052	500.077	499.970
8	499.874	499.983	499.996	500.046	499.855
9	499.911	499.982	499.994	499.992	500.028
10	499.889	499.959	500.018	500.008	499.992
Ave	499.881	499.963	500.024	500.045	499.946
Abs	−0.119	−0.037	0.024	0.045	−0.054
Std	0.024	0.022	0.021	0.050	0.061
Range	0.078	0.082	0.058	0.162	0.193
